# Polymeric Materials Used in 3DP in Dentistry—Biocompatibility Testing Challenges

**DOI:** 10.3390/polym16243550

**Published:** 2024-12-19

**Authors:** Florentina Rus, Cristina Neculau, Marina Imre, Florentina Duica, Alexandra Popa, Radu Mihai Moisa, Bianca Voicu-Balasea, Radu Radulescu, Alexandra Ripszky, Razvan Ene, Silviu Pituru

**Affiliations:** 1 Department of Biochemistry, Faculty of Dental Medicine, “Carol Davila” University of Medicine and Pharmacy, 8 Eroilor Sanitari Blvd, 050474 Bucharest, Romania; florentina.rus-hrincu@umfcd.ro (F.R.); alexandra.popa@drd.umfcd.ro (A.P.); mihairadu.moisa@drd.umfcd.ro (R.M.M.); radu.radulescu@umfcd.ro (R.R.); alexandra.ripszky@umfcd.ro (A.R.); 2 Faculty of Dental Medicine, “Carol Davila” University of Medicine and Pharmacy, 17-23 Calea Plevnei, 010221 Bucharest, Romania; cristina.neculau@drd.umfcd.ro; 3 Department of Complete Denture, Faculty of Dental Medicine, “Carol Davila” University of Medicine and Pharmacy, 17-23 Calea Plevnei, 010221 Bucharest, Romania; marina.imre@umfcd.ro; 4 Clinical Emergency Hospital Bucharest, Floreasca 8, 014451 Bucharest, Romania; 5 The Interdisciplinary Center for Dental Research and Development, Faculty of Dental Medicine, “Carol Davila” University of Medicine and Pharmacy, 17-23 Plevnei Street, 020021 Bucharest, Romania; bianca.voicu-balasea@drd.umfcd.ro; 6 Orthopedics and Traumatology Department, “Carol Davila” University of Medicine and Pharmacy, 8 Eroilor Sanitari Blvd, 050474 Bucharest, Romania; 7 Department of Professional Organization and Medical Legislation-Malpractice, “Carol Davila” University of Medicine and Pharmacy, 17-23 Plevnei Street, 020021 Bucharest, Romania; silviu.pituru@umfcd.ro

**Keywords:** 3D printing in dentistry, polymers, residual monomers, temporary prosthetic restorations, biocompatibility of 3D-printed materials, dental materials, biocompatibility tests, viability, cytotoxicity

## Abstract

In the latter part of the 20th century, remarkable developments in new dental materials and technologies were achieved. However, regarding the impact of dental resin-based materials 3D-printed on cellular responses, there have been a limited number of published studies recently. The biocompatibility of dental restorative materials is a controversial topic, especially when discussing modern manufacturing technologies. Three-dimensional printing generates the release of residual monomers due to incomplete polymerization of materials and involves the use of potentially toxic substances in post-printing processes that cannot be completely eliminated. Considering the issue of biocompatibility, this article aims to establish an overview of this aspect, summarizing the different types of biocompatibility tests performed on materials used in 3D printing in dentistry. In order to create this comprehensive review, articles dealing with the issue of 3D printing in dentistry were analysed by accessing the main specialized search engines using specific keywords. Relevant data referring to types of materials used in 3DP to manufacture various dental devices, polymerization methods, factors affecting monomer release, cytotoxicity of unreacted products or post-curing treatments, and methods for assessing biocompatibility were analysed. Although the introduction of new restorative materials used in dental treatments is subject to national and international regulations and standards, it is necessary to investigate them regarding biocompatibility in order to support or deny the manufacturers’ statements regarding this aspect.

## 1. Introduction

Dental medicine has a very long history; the first data on dentistry dates back to antiquity. Since ancient times, mankind has been concerned with the functionality, integrity, and appearance of teeth. If the papyri and mummies discovered by archaeologists dating from ancient Egypt show us that dental restorations were made by tying teeth or other materials such as ivory to the patient’s viable teeth with a gold wire, today’s technology has advanced so much that the patient’s teeth can be restored with great fidelity to regain both functionality and the desired appearance [1].

An extraordinary leap was made when 3D printing technology appeared in the medical field. Although this technology is already more than twenty years old in the medical field, the first kidney prototype printed in this way took place in 2002; in dentistry, it became interesting for researchers in 2010. In 2012, with the help of this technology, the first jaw was printed [2,3].

This progress has made dentists consider this technology in their field of activity, considering the numerous advantages brought into practice in the dental office, as well as in increasing the performance and speed of the medical act [3].

The additive technique, as the 3D printing technique is also called, can be applied in many specialities of dental medicine such as prosthodontics, endodontics, orthodontics, or even oral and maxillofacial surgical procedures [4]. Using this technique, dental components, surgical guides, braces with different uses, dental veneers, orthodontic devices, and provisional prosthetic restorations such as crowns or bridges can be made [4,5,6].

The first step towards the use of 3D printing technology in dentistry was the replacement of plastic imprints with intraoral scanning technology [7]. This further led to the obtaining of computerized digital models by using the CAD/CAM (computer-aided design/computer-aided manufacturing) technique [7,8]. This process first involves the acquisition of data through an imaging method and the transmission of this data to software that will analyse it, process it, and create the desired design (CAD). The data will be forwarded to another piece of software that is attached to a 3D printer, with the latter being responsible for making the desired model (CAM), reaching the so-called digital workflow [7,8].

Currently, the classic method is used in dentistry, that of milling from industrially polymerised blocks, which, until the final stage, is similar to the 3D printing method following the previously mentioned steps. Unfortunately, this method has some disadvantages: The production cost of the dental parts is higher, the equipment used is more expensive, the material losses are greater, and the production time of the dental parts of interest is longer. Taking into account these disadvantages and keeping pace with the progress of the scientific and technological world, the transition is made step by step towards a much more precise method: that of 3D printing, which benefits from numerous advantages [9,10,11].

First of all, the additive method is much more precise, thus generating much more precise dental materials from a physiological point of view [9,10,11].

Considering the fact that this method uses material that is added layer by layer to create the desired dental piece, the amount of residual raw material is reduced to a minimum, ultimately leading to a lower production cost. The resins used in 3D printing in dentistry are also very diverse, with numerous manufacturers putting such materials on the market with different properties depending on the part to be printed. This fact represents another advantage because, at this moment, the specialist has a fairly wide range from which to choose the best solution for their patients. Given such a vast range of resins, they do not have a high price either, and there is significant interest in this direction. The producers are careful to always bring new, clinically appropriate materials to the market [10,11].

In recent years, numerous models have appeared on the 3D printer market that can be used in dentistry. The technology is no longer so new and unknown, and this fact has attracted a decrease in the price of this equipment, contributing to a better production cost of prosthetic parts in dentistry. Currently, on the market, there are several types of 3D printers classified according to the manufacturing process of the final product: extrusion printing, inkjet printers, laser melting/sintering printers, and lithographic printers [12,13]. Another advantage is represented by the time required for 3D printing of a dental prosthetic piece, which is significantly reduced compared to the classic milling method. Using a faster and more precise technique, the dentist can make the medical act more efficient and much more oriented towards the patient, benefiting from a much more personalised treatment [10,11,12].

Although these advantages seem extremely attractive and progress has been accelerated in recent years, another essential aspect must be taken into account: the biocompatibility of the materials used in 3D printing [14,15].

Biocompatibility, in theory, is defined as the ability of a material to fulfil the proposed role at the level of an organism without acting on its state of health and balance [16]. Until recently, this aspect of biocompatibility referred to cell viability and toxicity [16]. According to EN ISO 10993-5 [17], biocompatibility tests refer to the proper use of medical devices in order to provide insights into the potential biological risks associated with the utilisation of dental prostheses and orthodontic devices. These tests follow the assessment in vitro or in vivo (cruelty-free) to detect the possible adverse reactions of devices that come into contact with the human body for a period of time. Evaluation of acute, sub-chronic, and chronic toxicity due to potential contamination with production residues following manufacturing procedures, post-manufacturing treatments, or foreign substances is conducted through several tests, such as the detection of cytotoxicity, genotoxicity, hemocompatibility, epicutan testing, endotoxin detection, and pyrogen detection [11,18].

The most commonly used materials in 3D printing techniques in dentistry for temporary prosthetic restorations are polymerizable resins, especially those based on methyl methacrylate or composite resins [14,15,19,20,21,22,23,24].

**Figure 1:**
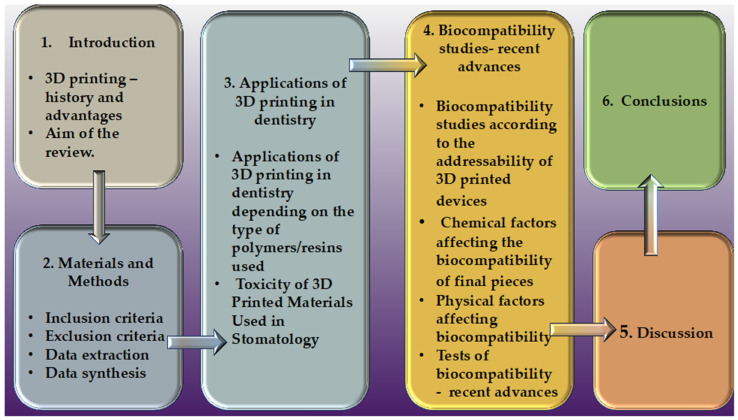
Review information flow.

Considering the issue of biocompatibility, this article aims to establish an overview of this aspect, summarizing the different types of biocompatibility tests performed on materials that are used in 3D printing in dentistry. This aspect aids both the doctor and the patient, leading to an increase in the quality of medical performance and to personalized, individualized, patient-oriented medicine. To navigate this review more easily, the informational flow can be seen in Figure 1.

## 2. Materials and Methods

This article aims to establish an overview of the biocompatibility of 3D-printed dental resins published to date. Our main purpose is to summarize the biocompatibility tests performed in vitro (laboratory settings) and in vivo (on living organisms) that investigate the biocompatibility of 3D printed dental resins, reporting on the interplay between the chemical composition, physical properties, and biological response of 3D-printed dental resins.

In order to create this comprehensive review, articles dealing with the issue of 3D printing in dentistry were analysed by accessing the main specialized search engines: PubMed/MEDLINE, Google Scholar, NCBI, Scopus, Web of Science, and Cochrane Library. Keywords such as “PMMA”, “polymers”, “polymethyl methacrylate”, “biocompatibility”, “cell toxicity”, “cell viability”, “stomatology”, “3D printers”, “3D printing in dentistry”, “residual monomers”, “temporary prosthetic restorations”, “PMMA crowns and bridges”, “biocompatibility of 3D printed materials”, “dental materials”, “washing solution”, “ethanol”, “post curing temperature”, “degree of conversion”, “unreacted monomers”, “biocompatibility tests”, “viability”, and “cytotoxicity” were used.

Inclusion criteria: Articles written in English, based on their relevance to the topic of dealing with the issue of biocompatibility testing performed on the materials used in 3D printing in dentistry, were analysed.

Exclusion criteria: Articles for which we did not have access to the full text, articles that referred to the use of 3D printing techniques in other medical specialties, and studies that dealt with materials other than those used in dentistry were excluded from the study.

Data extraction: Relevant data were extracted from the selected articles. Among these, data referring to types of materials used in 3DP to manufacture various dental devices, polymerization methods, factors affecting monomer release, cytotoxicity of unreacted products or post-curing treatments, and methods for assessing biocompatibility were used.

Data synthesis: The extracted data were analysed and synthesized in order to provide a comprehensive overview of the current knowledge regarding the biocompatibility of materials used in dental applications.

## 3. Applications of 3D Printing in Dentistry

The resins used in 3D printing techniques in dentistry are very diverse, with numerous manufacturers putting such materials on the market with different properties depending on the part to be printed [5,6,7,8,9,10,11,12].

The most commonly used materials in the 3D printing technique in dentistry for temporary prosthetic restorations are polymerizable resins, especially those based on methyl methacrylate or composite resins [5,6,7,8,9,10,11,12,13,14,15,16,19,20,21,22,24].

Classification of 3DP technology used in medical fields can be made according to different working principles, process parameters, and material composition, with the main printing technologies being powder bed fusion (PBF), light curing, and fused deposition modelling (FDM), each with its distinctive advantage regarding their application in dentistry (especially in the fields of prosthodontics and oral implantology) [13,14,15,16]. In recent years, numerous studies have been conducted on resins based on methyl-methacrylate used in 3D printing techniques, and the results obtained have been controversial. It has been observed that, in the case of these materials, a certain amount of residual unreacted monomers remains [11,13,14,15,16,19]. Additionally, it has been demonstrated that, over time, mechanical degradation, chemical interactions with the salivary enzyme system as well as with the bacterial system act on these temporary prosthetic parts made of resins, leading to the dilution of the remaining unreacted compounds trapped in the meshes of the resin network, all of which lead to the release of monomers [11,13,14,15,16,19,25,26,27,28,29,30,31,32].

### 3.1. Applications of 3D Printing in Dentistry Depending on the Type of Polymers/Resins Used

The main advantage of this 3DP technology is represented by the possibility to print some of the melting metals largely used in dentistry such as magnesium, cobalt-chromium, or titanium due to improved resistance and their biocompatibility [33,34,35,36].

Temporary or permanent dentures, crowns, and dental bridges can be manufactured from a multitude of materials through 3DP printing technology, but choosing the most suitable material and manufacturing process for a particular application can be challenging [13,25,27,37,38,39,40].

Some parameters of printable materials used in 3DP technologies applied in dentistry are material properties (not all printable materials are suitable for all dental applications), degree of accuracy in the reproduction of anatomically compatible objects (although 3DP can produce objects with a high level of accuracy, the final version of these objects could be influenced by some post-printed factors or by the resolution of the chosen model of 3DP and by the quality of the 3D model used as reference) [27,37,38]. Additionally, time, costs, and some regulatory considerations have to be considered when some types of materials are chosen to be used in 3D-printed dental object manufacturing to be utilized in clinical practice because they may need additional testing to demonstrate their safety and effectiveness [37].

Among the materials used in stereolithography, the main additive manufacturing technologies in the dental field are acrylate photopolymer, plastic, and ceramic due to their property that allows high-speed production of functional parts such as anatomical models, prosthetics, and various metal casting, but the limitations of this method includes fragility and a high cost of production [41,42,43,44]. Other 3DP processes applied in dentistry use different materials such as thermoplastics, powders, metals, ceramics, poly-carbonate, acrylonitrile butadiene styrene (ABS), polypropylene, polyesters, different metals and alloys like cobalt, steel, bronze alloy, aluminium, stainless steel, titanium, nickel alloy, other metal powders [41,42,43,44].

Some of the materials are commonly used in 3D printing to manufacture dental pieces due to their ability to bond and interact with living tissue and adaptable mechanical and degradation properties [41,42,43]. Among these, the most commonly used are several synthetic polymers and various resins, such as poly (ethylene glycol) (PEG), poly (vinyl alcohol) (PVA), polylactic acid (PLA), acrylonitrile butadiene styrene (ABS), ceramic and metallic alloys, biocompatible materials per se or in composite combinations [16,43]. The biocompatibility of all these materials was tested by evaluating the morphology and cell migration as markers of cytotoxicity in cell cultures, both for the objects manufactured by the classic CAD-CAM technique and for those obtained by using novel 3DP techniques [16,41,43].

The 3DP additive manufacturing (AM) process is widely employed in various fields of dentistry and has many advantages, but also some limitations. This method uses material that is added layer by layer to create the desired dental piece, such as endoprostheses, temporary dental crowns, epenthesis, endoluminal stents, maxillofacial guides, treatment templates, and bespoke repairs [16], as enumerated in Table 1.

For materials used in dentistry, there are several factors that will influence hardness values, and these include the time and speed of elastic recovery, the type of material printed, and the final shape of the manufactured part. Viscoelastic deformation depends on the type of printer used and the settings used, as well as the desired final result (type of printed part). Furthermore, there may not be a correlation between hardness values when comparing different indenter shapes. A universal hardness test, known as the Martens test, is suitable for testing the durability of most solid materials. It includes both elastic and plastic deformation effects and viscoelastic effects, depending on the type of printer used and the type of material used to print the designed parts [45,46,47,48,49,50,51,52,53,54,55,56,57,58,59,60,61,62,63].

Complete denture prosthetics and artificial teeth are among the main oral prosthetic devices produced nowadays by the 3DP technique, using a variety of photosensitive 3D resins and specifically engineered materials that are used by clinicians in current dental treatments in order to repair or to replace injured and diseased tissues [64,65].

### 3.2. Toxicity of 3D-Printed Materials Used in Stomatology

Toxicity is associated with diverse issues, such as mucosal irritation and the appearance of inflammatory processes, due to the cytotoxicity of uncured, residual, or unreacted monomers, a low monomer/polymer ratio, the degree of polymerization, the volatile nature of monomers, or the dusty nature of fine PMMA particles resulting from some of the procedures [29,32,37,66,67,68,69]. The resins used in the manufacture of objects used in various dental treatments are obtained following polymerization reactions, in which monomers are transformed into stable polymers by means of an addition reaction, using various sources of heat, light, or chemicals as activators [70,71]. These reactions generate toxic products, such as residual monomers and other toxic chemical waste products like formaldehyde, methyl methacrylate, benzoic acid, methacrylic acid, or dibutyl phthalate within the material, which can diffuse into the oral cavity, consequently generating cytotoxic effects on the oral mucosa [70,71,72]. A limited number of studies have shown that residual monomers and additives resulting from 3D printing techniques, milling, or conventional resin processing technologies used in the manufacture of splints required for various dental treatments could have a potential cytotoxic effect in contact with mucosal cells, gums, and in the presence of salivary enzymes and microbiota in the oral cavity [66,70,71,72,73].

The manifestations of cytotoxicity of this residual material are allergic reactions, extending to hypersensitive asthmatic responses, oral mucosa irritation, pulp injury, gingivitis, and periodontitis, as well as other general issues, such as conjunctivitis or neurological responses [73,74,75,76]. The cytotoxicity of objects used in dental treatments is influenced by the amount of residual substances, especially residual monomers, which interact with the tissues with which they come into contact. The residual monomers are obtained due to the incomplete polymerization of the materials used, depending on the method used in the manufacture of the resin [38,73,74,75,76]. The manufacture of dental prostheses requires the utilization of materials that present good mechanical and physical properties. Such materials are polymer-based materials, like various resins and composite resin-based materials, pure zirconium, or titanium [55,56,57]. Furthermore, ceramic or composite materials are used, or combinations of ceramics with other materials for 3D printing of various objects intended for dental treatments. Such materials include zirconium, aluminium, hydroxyapatite, and their combinations with thermoplastic polymers or various other metals, thus obtaining final pieces with increased durability and an appropriate aesthetic for applications in the dental field [41,77,78,79]. A challenge that must be considered when manufacturing dental prostheses through the 3D printing technique is the surface finish, as it must be smooth and polished on the surface to provide comfort and proper aesthetics during use [41,77,78,79].

In this context, the safety of using these new and modern devices is extremely important, although in the specialized literature, studies on the biocompatibility of the materials used in their manufacture, and especially the influence of post-printing processing treatments on biocompatibility, are limited.

## 4. Biocompatibility Studies—Recent Advances

In recent years, numerous studies have been conducted on resins based on methyl methacrylate used in 3D printing techniques, and the results obtained have been controversial. It has been observed that, in the case of these materials, following the steps performed by 3D printers, a certain amount of residual unreacted monomers remains [14,15,19,20,21,22,23,24].

In order to approach the definition of biocompatibility, researchers are working on optimizing the factors that influence the biocompatibility of materials used in 3D printing, such as washing time, washing solutions, and additional post-curing time, in order to eliminate residual monomers without affecting the physical, chemical, and mechanical properties of dental parts [18,25].

Although there are numerous aspects when we refer to the term “biocompatible” depending on the intended use, in accordance with the EN ISO 10993 [17,80,81] guidelines, biocompatibility tests refer to the proper usage of medical devices in order to provide insights into the potential biological risks associated with the utilization of dental devices over a period of time [18,25].

The materials used in 3DP must be certified to remain chemically stable for the intended period and not cause any adverse immunological response in order to be considered biocompatible [18,25].

Biocompatibility safety is essential in biomedical applications due to the interaction between living tissues that are in direct contact with various materials used in the production of different objects and devices, for a period of time, depending on their application. The health and safety concerns, as well as the general hazards associated with 3D printing technologies utilized in dentistry, are related to the specific processes and materials used [13,26]. The main aspects of biocompatibility studied regarding prosthetic provisory materials used to manufacture dental prostheses, dental crowns, and bridges were tested in cell cultures, where cell viability and the toxicity of the used materials were reported [13,26].

The utilization of biocompatible materials in the manufacture of a diversity of objects utilized in dentistry applications is very important. For example, the utilization of calcium phosphate-based materials as a reactive component in the powder binder printers (PBP) method is preferred due to their similarity to dental sources in creating implants [27,28].

Another example includes biocompatible clear plastic materials utilized to produce dental surgical guides and aligners in dentistry that need to be suitable for long-term wear in the mouth [28,29,30]. Biocompatibility in oral dentistry refers to an appropriate host response to specific dental treatments that are used in various prosthetic or orthodontic devices for a period of time, and the ability of the materials that were used in manufacturing processes to coexist with the host [5,27,28].

3DP technologies offer numerous advantages in the manufacturing processes of temporary (provisory) dental prostheses, crowns, and bridges compared with traditional wax loss technology and subtraction computer numerical control methods [29,30,31]

One advantage of CAD-CAM-3DP technologies (or additive manufacturing) used nowadays to design and produce three-dimensional objects to be used as medical devices in dentistry, for a period of time or permanently, is represented by the possibility of manufacturing a variety of shapes with almost unlimited levels of complexity that fit biologically almost perfectly with minimum post-printing modelling finish [29,30,31]. Another advantage is the possibility of choosing the most suitable material to be used in order to meet the needs of patients, as they could benefit from (more specific) personalized dental treatments. Additive manufacturing methods in dentistry use 3DP and laser melting processes to build objects by adding different materials layer by layer with differences in the way each layer is deposited and processed, but using these technologies can nevertheless influence the biocompatibility of final pieces [29,30,31].

### 4.1. Biocompatibility Studies According to the Addressability of 3D-Printed Devices

The biocompatibility of final devices is influenced by a number of factors, mainly by the type of material (resin matrix, resin composite materials, resin-based polymers), the 3DP technology used for manufacturing various objects, printing parameters, and the post-printing process, all of which are related to the quantity of residual components leached [64,82].

Biocompatibility is influenced by several factors. Depending on the intended use of the printed dental part, there are different materials, such as those based on silicone polymers or polyurethanes, that have the advantage of being completely cured without the need for a subsequent post-processing stage [83]. However, other types of materials, such as acrylic resins, are quite frequently found in dentistry. In order for them to achieve their physical, chemical, and mechanical properties, they need an additional processing stage and exposure to UV so that the material can cross-link the residual monomers, thus contributing primarily to achieving the ideal properties of the material and secondarily maximizing the biocompatibility of the material [84,85,86]. Another factor that must be taken into account when we talk about these types of resins used in 3D printing for temporary prosthetic devices dentistry is the washing process. This is not considered a complicated process, but it has a particularly important role in the elution of unreacted monomers [84].

### 4.2. Chemical Factors That Affect the Biocompatibility of Final Pieces

The chemical formulation of composite resins and the release of residual materials formed during the fabrication process or after post-fabrication in the final stages of finish the desired objects could lead to moderate or severe inflammatory reactions, in addition to degradation of physical and mechanical properties, such as poor bond strength, degradation, increase in water absorption rates, solubility, and the leachability of cytotoxic components from the resins [87]. As a result, the integration of new technologies, techniques, and instruments, together with the use of newly developed combinations of various resins, such as monomers of PMMA with oligomers, photo-initiators, nanoparticles, or other printable materials, has increased the advantage for allowing the fabrication of customized medical products with high-resolution and complex geometrical structure, improved mechanical properties, aesthetics, and relatively lower toxicity [41,88].

The chemical modification of various resins and composite materials used in 3DP manufacturing of dental objects depends on various process steps, such as printing settings, which vary according to the geometry of the designed parts, the orientation of the print, expected mechanical properties, biocompatibility, and duration of use in oral cavity conditions [88].

Recent studies have reported chemical modifications that were observed by the addition of dye into PPMA resin in order to improve the translucency and colour stability. These modifications affected the reaction kinetics in acrylic resins by slowing down the conversion reaction as the dye concentration increased. This reaction could be increased by adding 4,4′-bis (N,N-diethylamino) benzophenone to the resin as a co-initiator, resulting in a much higher conversion rate [88]. The polymerization reaction of PMMA (IUPAC name: poly [1-(methoxycarbonyl)-1-methyl ethylene]), a synthetic polymer obtained from the monomer methyl methacrylate (C_5_O_2_H_8_), transforms into polymethyl methacrylate (C_5_O_2_H_8_)n through activation under various conditions, which have cytotoxic adverse reactions due to the generation of free radicals upon heating [11,88]. By combining PPMA with other materials, such photo-initiators and oligomers with various chemical properties, dental resins such as UDMA, Bis-EMA, and TEGDMA there were developed with improved and suitable properties for use in clinical settings [89,90].

### 4.3. Physical Factors That Can Affect Biocompatibility

Three-dimensional printers have polymerization steps after each layer of resin is added, but studies have concluded that a final stage of post-curing temperature, carried out by exposure to UV light, would make these resins attain excellent mechanical, physical, and biocompatible properties by helping eliminate unreacted residual monomers. Through this stage, they are caught in the eyes of the previously formed networks [91,92].

Studies that indicate that this final stage of polymerization depends on many factors, including the composition and colour of the resin used, the UV exposure time, the type of curing room, as well as the intensity and frequency of the UV light [93]. These studies primarily investigated the physical, chemical, and mechanical properties of 3D-printed parts and came to the conclusion that a post-curing stage of 6 h at 140 degrees Celsius led to an improvement in the physical properties of the printed part [94,95]. However, Bayarsaikhan, E. and collaborators examined how biocompatibility, degree of conversion, and flexural strength were affected at different temperatures in the post-curing stage as well as at different exposure intervals. They concluded that flexural strength increases with increasing exposure times and temperatures [87]. Another parameter that was measured was the degree of conversion, and the results showed that, after an exposure of 120 min at 60 degrees Celsius, this had the highest value [83,87].

Regarding the issue of biocompatibility, better viability results were obtained, along with lower cytotoxicity, in the case of exposure to a higher temperature for a longer period of time [93,96].

In the case of polymerization of the resins used in 3D printing, a layer of unreacted monomers remains on the surface of the parts obtained [90]. Several studies have implicated these residual monomers in the cytotoxic potential of polymeric materials. Additionally, these unreacted monomers may generate an inflammatory response in the event that the dental piece comes in direct contact with dental tissues or induce mitochondrial damage [97,98,99,100,101]. Furthermore, these residual monomers favour the multiplication of plaque-forming microorganisms [102].

In order to prevent these effects with negative potential, it was concluded that this layer of unreacted monomers should be removed. This is easily achieved by washing post-polymerization with alcoholic solutions. Although this seems simple in theory, it is more complex in practice because not every alcoholic solution can be used at this stage [103,104,105]. Thus, studies have shown that if ethanol or even a solution of ethanol with water is used, it will affect the physicochemical and mechanical properties of the parts printed from resins based on methyl methacrylate. These parts will have lower resistance, so cracks will appear on their surface, or they will dissolve to a certain extent [103,104,105]. Thus, studies were carried out focusing on the type of alcoholic solution used at this stage, as well as the duration of this washing. One of these studies is the one carried out by Na-Kyung Hwangbo and his collaborators, in which washes with isopropyl alcohol (IPA) and tripropylene glycol monomethyl ether (TPM) were compared [106]. The duration of this washing stage varied from 3 to 90 min, and the results showed that there were no significant differences between the two washing solutions used, but significant differences were obtained regarding the duration of exposure of the polymerized material to the alcoholic solutions [106]. Thus, the increase in the washing period was associated with an increase in cell viability, as well as a decrease in the cytotoxic effect of the residual monomers [106].

It was also observed that none of the substances used for washing, regardless of the nature of the treatment, led to physicochemical or mechanical changes to the material, both its structure and properties remaining as good [42]. Other ways of washing this layer of unreacted monomers involved washing with IPA along with ultrasonication for 5 min; these also had good results both from the point of view of biocompatibility and the integrity of the material structure and the preservation of mechanical properties [84,88]. 

On the other hand, if the duration of this washing is longer than 12 h, it leads to damage to the polymer network, with the solvent penetrating the surface of the matrix of the polymerized material [84]. Washing solutions such as acetone and butyl glycol have also been proposed to remove the layer of monomers with cytotoxic potential, but studies have shown that these solvents are unsuitable because they weaken flexural strength [11].

### 4.4. Tests of Biocompatibility Recent Advances

In the scientific literature that reports the biocompatibility of 3D-printed dental resins and the fabrication parameters that can influence biocompatibility with oral tissues, the existing body of evidence is quite limited [70,107].

Some studies reported only manufacturer-stated certification that was taken into consideration prior to the use of a variety of specifically engineered biocompatible materials, photosensitive 3D resins for manufacturing endoprostheses, temporary dental crowns, epenthesis, endoluminal stents, maxillofacial guides, treatment templates, and bespoke repairs [16,71].

Depending on the type of polymers chosen and the 3DP technique used, acrylates and their esters are the most commonly used resins in dentistry as filling and replacement materials [79,108]. Other industrially usable materials for the fabrication of dentistry devices are mentioned in diverse scientific studies, but tests on the cytotoxicity of final pieces or on the biocompatibility of the various pieces used in direct contact with oral tissues, in vivo or in vitro, are limited. In a study conducted by Tzeng, J.-J. and co-workers (2021), the cytotoxicity level of five urethane acrylates (UAs) was evaluated in comparison with a commercial 3DP denture base acrylic resin (control group) by measuring the level of MTT. The conclusion was that the UA-based photopolymer resins were nontoxic [41,45]. The UA-based photopolymer resins reported in this study were aliphatic UA (588), aliphatic urethane hexa-acrylate (87A), aliphatic urethane triacrylate diluted in 15% HDD (594), aromatic urethane hexa-acrylate (88A), and high-functional aliphatic UA (5812), which were prepared for digital light processing (DLP)-based 3D printing. They were mixed with ethoxylated pentaerythritol tetraacrylate (40 wt%), isobornyl acrylate (12 wt%), diphenyl (2,4,6-trimethylbenzoyl) phosphine oxide (3 wt%), and a pink acrylic (5 wt%), and further processed to make specific pieces that were evaluated for mechanical properties and cytotoxicity. The conclusion was that the UA-based photopolymer resins were nontoxic [45].

Polymers used for additive processes in dentistry that are employed in polymerization processes such as digital light processing (DLP), polymer jetting (MJ), and laser stereolithography (SLA)—all of these methods having in common the process of material extrusion (ME) using fused deposition modelling (FDM)—according to a study by Jockusch, J. and collaborators (2020), have different composition [108]. For example: vinyl polymers, e.g., polyvinyl alcohol (PVAL); styrene polymers, including polystyrene (PS) and acrylonitrile-butadiene-styrene (ABS); acrylates, which are polymers of acrylic acid or methacrylic acid and their esters, processed by polymerization reaction; polyesters (saturated, linear, or thermoplastic condensates)—polycarbonate (PC) in combination with various functional additives like phosphites, phosphines, and 2-hydroxybenzophenones, used to improve mechanical properties of final products; polyamides (PA) used in combination with functional, filling, and reinforcing additives, for example, polyamide 11/12, thermoplastic polyamide elastomers, and nylon/PA 66; polyether ketones—polyether ether ketone (PEEK)—that could be used for multiple applications due to their good mechanical properties and high in vitro biocompatibility (non-cytotoxicity) [73,109,110,111,112]; polyadducts, such as epoxy resin (also epoxy or ethoxylin resin; EP); natural polymers/bio-polymers, such as polylactide (also polylactic acid, PLA) [113]; and biocompatible supramolecular polymers (PCL) and their composites [108,113,114]. These polymers met the ISO 10993-5 [17] and 10993-12 [81] standards for cytotoxicity and biocompatibility according to Jockusch, J.’s study (2020), although polyether ketones—polyether ether ketone (PEEK)—presented high in vitro biocompatibility. Moreover, the composition of PEEK polymers can be modified by the addition of various substances, such as hydroxyapatite, tricalcium phosphate, carbon, and glass fibre, in order to increase mechanical properties, while at the same time maintaining its biocompatibility properties [108].

Numerous other photopolymerizable resins are available, composed of multi-functional monomers based on methacrylate or acrylic esters [115], which are used to build a polymeric network in combination with various dyes or colorants added as photoinitiators to improve the physical and mechanical properties of 3DP specimens, mainly obtained by stereolithography (SLA) and digital light processing (DLP) [35,115].

The main drawback related to the use of photopolymerization techniques in the manufacture of dental devices is related to the availability of FDA-approved biocompatible materials; therefore, the need to test as many polymerizable resins as possible remains a challenge in the field of dentistry [36,115]. In Table 2, we summarize the main types of tests used to evaluate the degree of biocompatibility and cytotoxicity of 3DP devices used in dentistry, based on the materials used, manufacturing processes, and types of reported tests.

A study conducted by Pitzzanti and co-workers (2024) investigated photocurable resins based on urethane dimethacrylate (UDMA), combined with one of the following co-polymers: triethylene glycol dimethacrylate (TEGDMA), ethylene glycol dimethacrylate (EGDMA), diethylene glycol dimethacrylate (DEGDMA), and diphenyl(2,4,6-trimethylbenzoyl) phosphine oxide (TPO) as a photoinitiator [115]. The study evaluated the mechanical properties and biocompatibility of the obtained samples by measuring the cytotoxicity of the printed resin specimens in accordance with ISO 10993-5 [17]. They reported that changes in mechanical properties (depending on the water contact angle measurements) under tensile conditions could influence the suitability of the material for a specific biomedical application [115]. The water absorption capacity of resins (materials) is an important parameter to consider when it comes to obtaining a specific dental device that comes into contact with human tissue for different periods of time [115]. Hydrophilic surfaces are used to manufacture short-term implants due to their capacity to limit cell adhesion, a fact that could enhance biocompatibility. In contrast, hydrophobic surfaces enhance cell adhesion, making these types of materials suitable for the manufacture of devices used in tissue engineering and various other biomedical applications [115]. Pizzanti et al. explored the mechanical properties, water sorption capacity, biocompatibility, and cytotoxicity tests of resin mixtures [115]. Their study revealed promising results that might recommend these mixtures for use in different biomedical applications, not only in the dentistry field [35,36,102,115,116,117,118].

**Table 2:** Biocompatibility of 3DP devices used in dentistry as a function of the materials used, manufacturing processes, and types of tests use to evaluate the degree of biocompatibility and cytotoxicity.

Materials	Applications	Biocompatibility Tests	Influence on Biocompatibility of Diverse Processes in Manufacturing Techniques	References
Polymethyl methacrylate (PMMA) resin, ceramic, metals	Prosthodontics, orthodontics, orthognathics, endodontics, craniofacial, oral, and maxillofacial surgical procedures; manufacturing of implant-drill guides for guided surgery procedures	Cytotoxicity, genotoxicity, and irritation potential; regulation of standards USP Class IV and ISO 10993; cellular assays; cellular response; in vivo toxicity on model animals (zebrafish embryo, culture of mouse oocytes)	Exposure to UV so that the material can cross-link the residual monomers; role of the washing process in the elution of unreacted monomers; post-curing treatments; variation of temperature,	[13,16,17,18,24,25,26,42,43,44,65,72,73,74,75,76,80,82,119,120,121,122,123,124,125,126,127,128,129,130,131,132,133,134]
Acrylonitrile butadiene styrene (ABS), polylactic acid (PLA), polycarbonate (PC), polyamide (nylon), thermoplastic materials	Orthodontic alignment trays; retention trays or whitening trays, whitening trays; removable partial prostheses	Cellular assays; cellular response; viability and toxicity tests; cell viability; tissue integration; in vitro human cell adhesion and proliferation; MTT and XTT assays	Variation of UV light intensity and wavelength; variation of curing time and temperature washing solutions (isopropyl alcohol and tripropylene glycol monomethyl ether)	[13,16,18,24,25,26,27,33,45,73,74,75,76,80,83,84,85,86,88,108,109,110,111,119,134,135]
Polymethyl methacrylate (PMMA), thermoplastics (polycarbonate, polyamides, polyvinyl chloride) (meth)acrylate monomers, oligomers, photoinitiators, waxes	Dentistry and orthodontic applications; clear aligners; 3D-printed denture teeth; crown and bridge	Cell viability, cytotoxicity, and status of human gingival fibroblasts; confocal laser scanning microscopy	Addition reaction; using various sources of heat, light, or various chemicals as activators; washing post-polymerization with alcoholic solutions	[11,13,18,24,25,26,27,33,35,36,42,45,72,73,74,75,76,77,78,79,80,84,85,86,87,88,89,90,92,93,94,95,96,97,98,99,100,101,103,104,105,106,109,110,111,112,113,114,115,116,119,120,121,131,133,134,135,136,137,138]
Metal materials and alloys: titanium (Ti), titanium-based alloys: niobium, tantalum, zirconium, molybdenum, tungsten (Ti-Nb, Ti-Zr, and Ti-Mo-W); nickel-based and cobalt-based metal alloys:cobalt-chromium (Co-Cr)	Crafting biocompatible dental implants and orthodontic appliances	Evaluating morphology and cell migration; confocal laser scanning microscopy	Variation of UV light intensity and wavelength; variation of curing time and temperature	[13,16,18,24,25,26,33,34,40,41,77,78,79,102,107,108,115,117,118]
Ceramics, resin Ceramics, metals	Dentistry and orthodontic applications, clear aligners; clear orthodontic aligners; 3D-printed denture teeth; crown and bridge	Morphology and cells migration; Confocal laser scanning microscopy	Variation of UV light intensity and wavelength; variation of curing time and temperature; washing post-polymerization with alcoholic solutions	[13,18,24,25,26,30,31,32,33,34,38,72,73,74,75,76,83,123]
Ceramics: compounds of metallic elements and non-metallic substances (oxides, nitrides, and silicates), most commonly used silica (SiO_2_)	Crafting biocompatible dental implants and orthodontic appliances	Mass spectroscopy; morphology and cell migration; confocal laser scanning microscopy	Variation of UV light intensity and wavelength; variation of curing time and temperature; washing post-polymerization with alcoholic solutions	[13,18,24,25,26,30,31,32,33,65,80,83,135]

The disadvantages of manufacturing processes that can occur in all techniques and materials include lack of mechanical strength, limited quality, high cost, limitations in printing resolutions, lower strength, poor surface finishing, and the need for post-processing.

## 5. Discussion

When a dental device is placed in contact with the tissue and fluids of the oral cavity, regardless of the type of material used for its manufacture, there are invariably different interactions between the device and the biological environment in which it is placed.

Excellent physicochemical, mechanical, and, last but not least, biocompatibility properties are key features of a dental material that is suitable for different dental devices. In order to obtain these properties, the materials used in 3D printing have been, and are constantly being, improved, along with the technique of the equipment used. Depending on the manufacturing processes, the final devices obtained for use in clinical dental treatments can manifest various degrees of cytotoxicity due to unreacted monomers that could leak into the oral cavity [11,90,94,95,101,135].

Today’s technology has advanced so significantly that the patient’s teeth can be restored with great fidelity to regain both functionality and the desired aesthetics in the dental industry. For this purpose, several types of technologies and materials are used to manufacture suitable devices, applicable for various periods of time, depending on the targeted effect. The manufacturing of custom models that respect the patient’s anatomy, from bifunctional and biocompatible materials, is conducted mainly using two forming processes. These manufacturing processes can be physical (fusion followed by solidification, sintering) or chemical (polymerization reactions), in which monomers are transformed into stable polymers by means of an addition reaction, using various sources of heat, light, or chemical activators as activators [91,92,106]. In this manuscript, we analyse the most relevant studies regarding clinical research on the biocompatibility of different materials used in dentistry.

Some of the factors that influence the biocompatibility of materials used in 3D printing including washing time, washing solutions, and additional post-curing time.

**Figure 2:**
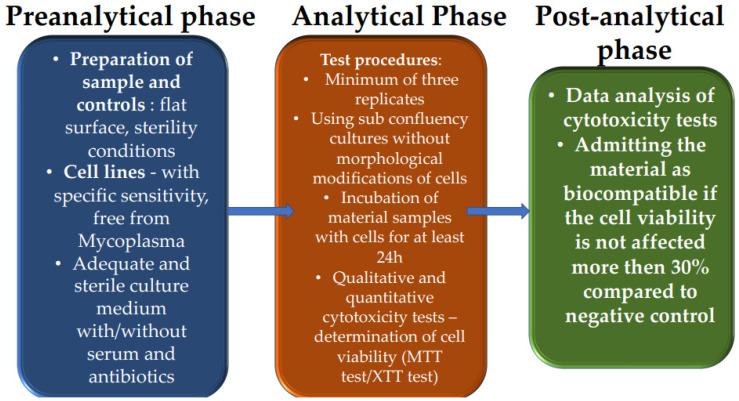
Steps to determine the biocompatibility of materials according to ISO 10993-5 [17].

Although there are numerous aspects when we refer to the term ‘’ biocompatible’’, in accordance with guides from the EN ISO 10993 [17] series, biocompatibility tests refer to the proper usage of medical devices to ensure their safe use and to reduce their potential to cause harm or adverse reactions. The main tests recommended by the EN ISO 10993 [17] series refer to cytotoxicity, sensitization, irritation, and genotoxicity, as seen in Figure 2 [137].

## 6. Conclusions

Although the technique of 3D printing in dentistry has gained momentum in recent years and numerous dentists have started equipping their clinics with 3D printers, the problem of residual monomers is still not fully elucidated. Reducing the amount of these residual monomers is attempted by adjusting post-processing steps, such as post-polymerization or post-processing washing.

Another impediment that stands in the way of the usual use of this technique in current practice is the biocompatibility testing of materials used in dentistry, which is currently limited to cell viability and cytotoxicity tests.

There are numerous intra- and intercellular biochemical mechanisms that can lead to damage or inflammation of gingival tissue. This is why more detailed biocompatibility tests are needed before materials used in 3D printing in dentistry can be used safely, which would be advantageous for both the patient and the dentist.

## Figures and Tables

**Table 1:** The common 3D-printed materials used in manufacturing various dentistry devices (pieces).

Tipe of Materials	Manufactured Pieces	Temperature Range Processing (FST)	Hardness Vickers and Martens Methods (N/mm^2^)	References
Synthetic polymers: Polyether ether ketone (PEEK), polycaprolactone (PCL), polymethyl methacrylate (PMMA), polylactic acid (PLA), poly(lactic-co-glycolic acid) (PLGA), and ultraviolet (UV) resins.	AM-printed occlusal appliances; Clear aligners; Crown or bridge copings; Implant abutments; 3D-printed denture teeth.	Between 58 °C and 430 °C, various periods of time depending on the designed pieces to be printed.	142–2926	[45,46,47,48,49,50,51,52,53]
Metals and metal alloys: Titanium (Ti) and cobalt-chromium (Co-Cr) alloys.	Implants, prosthetics, dental restorations.	900–2370 °C	44.3–286 GPa	[46,47,48,49,52,53,54,55,56]
Ceramics: Glass, zirconia, alumina.	Dental implants; Prosthetic implant abutments; Bridges; Root posts; Ceramic crown.	800–1600 °C	30–800 MPa 0.7–5 MPa·m _1/2_	[49,53,57,58,59,60,61,62,63]

(1) Conventional materials. PEEK: Polyether ether ketone. PMMA: Polymethyl methacrylate. PLA: Polylactic acid. PLGA: poly(lactic-co-glycolic acid). Ti: Titanium. Co-Cr: Cobalt-chromium. (2) FST: Final sintering temperature; (3) Elastic modulus, GPa; (4) Flexural strength (σ), MPa; (5) Fracture toughness (KIc), MPa·m _1/2_.

## Data Availability

Not applicable.
